# What Makes for Good Anesthesia Teaching by Faculty in the Operating Room? The Perspective of Anesthesiology Residents

**DOI:** 10.7759/cureus.2563

**Published:** 2018-05-01

**Authors:** Shin Wakatsuki, Pedro Tanaka, Rafael Vinagre, Adrian Marty, Jakob Louis Demant L Thomsen, Alex Macario

**Affiliations:** 1 Department of Anesthesiology, Perioperative and Pain Medicine, Stanford University School of Medicine; 2 Department of Anesthesia, Herlev and Gentofte Hospital, University of Copenhagen

**Keywords:** anesthesiology, graduate medical education, resident teaching

## Abstract

Background

Teaching during patient care is an important competency for faculty. Little is known about anesthesiology resident preferences for teaching by anesthesiology faculty in the operating room (OR). If the behaviors and characteristics of anesthesia teaching in the OR that are most valued by residents were identified, faculty could incorporate that best practice to teach residents during OR cases. The objective of this phenomenological study was to interview anesthesiology residents to determine what they perceive the best faculty teachers are doing in the OR to educate residents.

Methods

Thirty randomly selected anesthesiology residents (10 in each post-graduate year class) were interviewed using a semi-structured approach with a predetermined question: “Based on your experiences as a resident, when you think about the best-attending teachers in the OR, what are the best-attending teachers doing in the OR to teach that other faculty maybe are not doing?” Interviews were recorded, transcribed, converted into codes, and grouped into themes derived from the cognitive apprenticeship framework, which includes content, teaching methods, sequencing, and social characteristics.

Results

Resident responses were separated into a total of 134 answers, with similar answers grouped into one of 27 different codes. The most commonly mentioned codes were: autonomy – step back and let resident work through (mentioned by 13 residents), reasoning – explain why attending does things (12), context – teach something relevant to the case (8), commitment – take time to teach (8), literature – bring relevant papers (8), prior knowledge – assess the baseline level (7), flexibility – be open to trying different approaches (7), focus on just a few learning points (6), reflection – ask resident questions (6), provide real-time feedback (6), teach back – ask residents to explain what they were taught in their own words (5), belonging – facilitate communication with the OR team (5), psychological safety – be open and approachable (5), equanimity – stay calm and collected (5), select proper timing for instruction when the resident is not occupied with patient care (5), visualization – use graphs or diagrams (5), and specify learning goals ahead of time (5).

Conclusion

The best practice for OR teaching, as perceived by anesthesia residents, includes social characteristics, such as context, commitment, psychological safety, equanimity, and proper timing, as well as teaching methods, such as autonomy, reasoning, literature, prior knowledge, flexibility, reflection, real-time feedback, and teach back. Further studies can determine if training anesthesiology faculty to incorporate these elements increases the caliber of daily teaching in the OR.

## Introduction

Teaching is an important competency for academic faculty in graduate medical education because the optimal training of residents lays the foundation for high-quality patient care. In addition, academic faculty who score poorly on resident evaluations of their teaching are at risk for not being promoted and may seek counsel on how to improve their teaching.

The teaching of house staff can occur in various environments, such as the classroom, outside of direct patient care. In the classroom setting, for example, the following have been identified as useful: utilizing a clinical case vignette, clearly stating the goals of the talk, showing enthusiasm for the topic, reinforcing learning points, employing graphics or visuals, adding quizzes, providing recommendations about patient care, incorporating references/directions for further reading, eliciting active involvement of residents, and listing take-home messages [1-2]. However, the majority of graduate medical education involves work-related activities by the resident in the hospital or clinic delivering patient care [3]. Teaching in this setting may be challenging due to the need for teaching to occur simultaneously while both the resident and the attending have patient care work to do, the time constraints of faculty, the short stays of patients, and the absence of formal training in the education of the faculty. Desirable attributes for attendings include being clinically competent, clear, organized, accessible, supportive, compassionate, able to establish understanding with learners, provide direction and feedback, exhibit integrity and respect for others, offer a broad repertoire of teaching methods, engage in self-evaluation and reflection, and target teaching to the learners’ level of knowledge [4].

Anesthesia teaching by faculty for anesthesia residents in the OR during the care of an anesthetized patient has several challenges. Teaching occurs during the acute care of a patient that can rapidly change during difficult cases, production pressure exists to get through the list of OR cases in a timely manner, faculty are often covering two anesthetizing locations, and there is a need for both cognitive and technical skills instruction [5]. A study of faculty supervision evaluation by anesthesia residents identified nine key dimensions for the instructor: provides feedback, is available, gives opportunities/fostering resident autonomy, stimulates patient-based learning, demonstrates professionalism, is present during critical events, discusses the perianesthesia management of the patient, shows interpersonal skills, and exhibits concern for safety [6]. A separate study utilized the residents' written feedback to their clinical teachers to identify several themes associated with high teaching scores: use of primary literature to support teaching, explaining why specific management strategies were used, having education-oriented discussion, spending adequate time teaching, teaching to the appropriate level of the resident, demonstrating an active effort in teaching the resident, and imparting significant clinical knowledge [7].

If the teaching behaviors and characteristics of the anesthesiology faculty that are most valued by residents in the OR could be further elucidated, the anesthesiology faculty could incorporate these resident preferences for teaching during OR cases. Resident perception and preferences matters because physicians in anesthesia training are adult learners and, as such, should be involved in the planning and evaluation of their instruction. The perceived value that learners place on educational experiences is a measure of the learning environment and the learning environment can have a strong influence on the effectiveness of teaching.

The objective of this phenomenological study was to interview anesthesia residents to determine what the best faculty teachers are doing in the OR to educate residents.

## Materials and methods

The Stanford Institutional Review Board determined that this study did not meet the federal regulations’ definition of human subjects research and was exempt from review. This study employed a qualitative methods design. The Standards for Reporting Qualitative Research were followed [8].

A phenomenological approach was chosen because the goal was to describe the essence of a phenomenon (OR teaching) by exploring it from the perspective of those (anesthesiology residents) who experience it so as to understand the meaning they ascribe to OR teaching.

Sample size calculation was not performed for this qualitative study, as resident interviews were stopped when the responses no longer resulted in new data for new codes [9].

Using a random number generator, anesthesiology residents from each of the three resident classes at Stanford University Medical Center were selected from January 16 to March 3, 2017. All residents invited to participate agreed, and no incentives were offered for participating.

The participants were interviewed face-to-face by two investigators (S.W., R.V.) using a semi-structured approach with the aim to provide a supportive environment and encourage rich responses. Both investigators participated in all interviews. No written questionnaire was used. The participants were informed that the objective of the research was to understand their perceptions about what makes for good anesthesia teaching by faculty in the OR.

The wording of the main question was created during a meeting of the research team to narrow the research objective. This predefined question was: “Based on your experiences as a resident, when you think about the best-attending teachers in the OR, what are the best teachers doing in the OR to teach that other faculty maybe are not doing?”

If the resident gave a short answer of a few words or less, the resident was prompted with “Please tell us three things or more” as a way to signal that more answers were desirable and to get a more overall depth of content. When the resident was finished speaking, he/she was then asked, “Is there anything we haven't talked about that you want to add?”

The interview audios were digitally recorded and transcribed by the interviewing investigators without identifying data. The interview durations ranged from seven to 12 minutes, and all the interviews occurred in the anesthesia department offices in the hospital.

All de-identified responses remained anonymous. Initial transcripts were read in detail and fragments of the participants' responses were selected and then stored in a spreadsheet with other similar fragments. All six members of the research team sat in a classroom and reviewed and analyzed the fragments and responses to create codes. The participation of several researchers with varying perspectives helps provide multiple observations and conclusions, as this triangulation adds breadth and can facilitate a confirmation of findings.

Names for the codes, either a single word or a few words, were derived directly verbatim from the raw text. The general goal was for each code to be a specific behavior that could be easily understood and communicated to an interested faculty. Transcripts were examined multiple times as additional interviews were completed to provide ongoing comparisons across the dataset. A 100% coding agreement was reached by reviewing all code applications and discussing each disagreement until consensus by the team of authors was reached.

**Figure 1:**
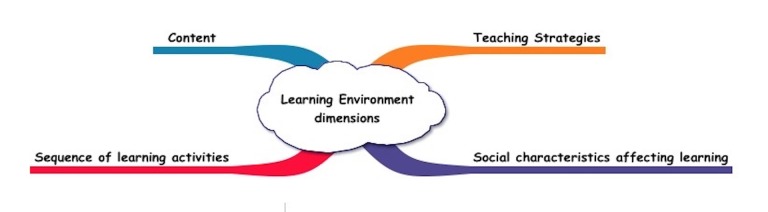
Cognitive Apprenticeship Framework of the Learning Environment

As the responses were converted to codes and as the number of codes increased, the research team renamed and reorganized codes, resulting in a grouping of codes by themes based on the cognitive apprenticeship framework, which has four themes: 1) teaching strategies (e.g., modeling, coaching, scaffolding, articulation, reflection, and exploration) for promoting expertise development; 2) social characteristics affecting learning (e.g., real-world context, practice communities, learner motivation, and cooperation among learners; 3) content required for expertise such as facts, concepts and procedures, and problem-solving, metacognitive, and learning skills; and 4) sequencing of learning activities, thereby increasing complexity and diversity and building global conceptual maps before acquiring specific skills (Figure 1) [10-11].

## Results

The responses by the 30 residents (12 male, 18 female) were separated into a total of 134 answers. Every resident listed a minimum of three answers because they were prompted that way, with most residents stating four or five different ideas. The answers were then grouped into one of 27 different codes (Table 1). None of the codes were deemed to fit into the cognitive apprenticeship framework theme of sequencing.

**Table 1:** Codes for All Resident Responses Grouped into Teaching Strategies, Social Characteristics, or Content ABA: American Board of Anesthesiology

	Code	Total number of times mentioned	CA-1	CA-2	CA-3
Teaching Strategy	Autonomy (step back & let resident work through)	13	3	6	4
	Reasoning (explain why attending does things)	12	6	3	3
	Literature (bring articles relevant to the case)	8	2	2	4
	Flexibility (open to trying different things)	7	2	2	3
	Prior knowledge (assess the baseline level)	7	1	5	1
	Real-time feedback	6	2	2	2
	Reflection (ask the resident questions)	6	0	3	3
	Teach-back (ask residents to explain what they were taught in own words)	5	0	1	4
	Visualization (use graphs or diagrams)	5	3	1	1
	Specify learning goals (before the start of the case or the day before)	4	2	2	0
	Discussion (use two-way communication)	3	1	1	1
	Breaking down a complex thing	2	1	0	1
	Practice techniques in non-pressure environment	2	1	1	0
	Multimodal teaching	1	0	1	0
	Real-time teaching	1	1	0	0
	Link teaching content to ABA exams	1	0	1	0
	Challenge out of comfort zone	1	0	1	0
Social characteristics	Context (teach something relevant to the case)	8	3	2	3
	Commitment (take time to teach, make effort)	8	4	2	2
	Psychological safety (be open and approachable to questions from resident)	5	2	2	1
	Equanimity (remain calm and collected)	5	2	1	2
	Proper timing for instruction	5	3	1	1
	Belonging (facilitate communication with OR team)	5	1	2	2
	Confident clinically	3	0	2	1
	Personable and open	3	2	0	1
	Ask for feedback for themselves	2	0	1	1
Content	Focus on a few learning points	6	3	1	2
	TOTAL	134	45	46	43

**Figure 2:**
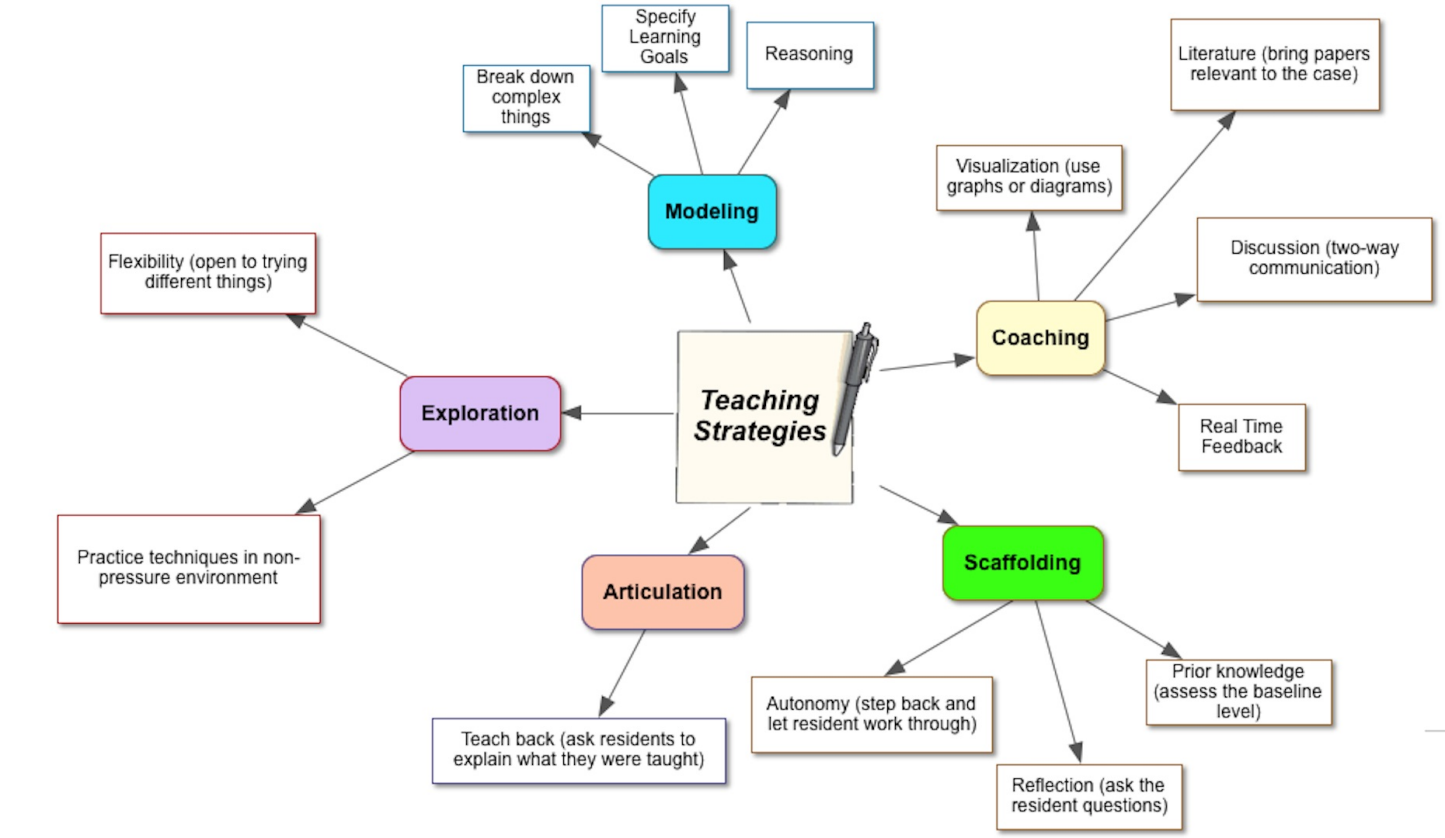
The Teaching Strategies Most Commonly Mentioned by Residents About the Best Faculty Educators

Teaching strategy codes were categorized into different cognitive apprenticeship framework types (Table 2, Figure 2).

**Table 2:** Teaching Strategy Codes with Sample Verbatim Quotes to Illustrate

Scaffolding – provide supports to encourage the performance of activities at their learning edge & gradually decrease support over time	
Autonomy (step back and let resident work through)	“[The best teachers] are really good at when to take a step back and allow the resident to work through their procedure or their line of thinking as opposed to jumping in and doing what needs to get done.”
	“They're comfortable knowing that they can rescue you and letting you, if you need to even struggle little bit, to accomplish a task. So, in that way, you can learn it better and appreciate it more. And they're not stepping in too fast to take over.”
Prior knowledge (assess the baseline level)	“The best teachers access knowledge before giving knowledge. They ask questions about the learner’s baseline understanding, and build up there and push the edge in that way.”
	“Really trying to gauge what level I am at. Instead of just automatically teaching something better trying to see what I already know. Asking a lot of questions and trying to gauge what level of learning I'm at so that they're really teaching to the level of the learner.”
Reflection (ask the resident questions)	“Faculty come to mind who really challenge you, wonder why you pick a certain drug or a certain type of anesthetic - what your reasoning is behind it. There might be evidence you have. Some residents may not like being challenged that way. I enjoy having somebody just keep asking, ‘Why do you do this?’”
	“The best teachers set me up with a problem. They say, 'OK. What if this happens right now, what are you going to do and why? What's your differential?' That way, it keeps you involved in the current case and also makes you think about things you end up learning.”
Modeling – let learner observe you perform the activity, think out loud explicitly demonstrate both physical act & thinking	
Reasoning (explain why attending does things )	“[The best teachers] explain what they do because everyone has a different style and how they do it. They provide a reason of why they do what they do. I understand there's a lot in anesthesia that hasn't been clarified completely regarding evidence based medicine . . . To develop one’s style and one's proper and good anesthetic care, it's nice to know their reasons. This way you can agree with those reasons and then talk about that technique. Or you can say I would like to adopt this technique but only when those reasoning apply to my patient.”
	“What I learn the most from the attendings is when I take these little things. This is why I put OG tube like this, this is why [I put the] temperature probe like this, this is why I bag-mask like this.”
	“The best teachers, they clearly have a strong understanding of physiology and a way to explain it. The best is when you get logical explanation that's backed up by science for why they're teaching you something. That's how I want to practice. That's what I want, how I want to decide what I want to do. Like it's good to see different preferences just based off of experience but I like to have reasoning backed up by science as to why I'm doing the things I'm doing.”
Specify learning goals (before start of case or day before)	“I prefer when they tell me the outline, for example, ‘We’re going to talk about three items today. Here is No. 1, No. 2, and No. 3. Let’s start No. 1.”
	“I would say, like, taking an element of the case, an equipment, a medication, or overall principle of anesthesia and, ‘This is what we are going to learn about today.’ So having purpose and a precise learning object is helpful.”
Breaking down a complex thing	“They explain things in such a way that they are not explaining to an equal level attending colleague but to someone who might be doing it for the first time. Even if it’s simple, . . . breaking it down and taking you through each step.”
Coaching – observe learner perform the activity and provide directed feedback and guidance	
Literature (bring papers relevant to the case)	“Bring papers and resources to discuss and review in the OR . . . [An attending] discussed how the study affects the way he manages all patients and especially our current patient. This is helpful because it provides concrete and specific evidence as well as discussion that makes me more likely to agree with and adopt the technique in the future.”
Real-time feedback	“Immediate feedback helps you change things . . . if your technique with intubation is like, 'It could be better this way. This is how you did it. This is how you should do it.' And you could practice on the next case. So it’s something you can fix real-time, I think that’s better than waiting around and being vague about it."
	“The thing that I like the most is someone who just teaches as you go along rather than like waiting to the end of the day or waiting till your evaluation months a later to tell about things that were done well and things that could be done better. I think it's just best to do it in real time especially since anesthesia is so hands-on . . . because I want to be able to improve now rather than waiting like a couple months”
Visualization (use graphs or diagrams)	“I like the people who do diagrams . . . they'll grab a piece of paper . . . break it down with diagrams and flowcharts . . . you trained to learn rather than just hearing it but also seeing their words or seeing the principles written down.”
Discussion (two-way communication)	“I prefer . . . teaching and application better than just being talked to. I’d like to do a discussion.”
Exploration – invite learners to identify and undertake new learning activities or pose and solve their own questions to promote independent learning	
Flexibility (open to trying different things)	“Be flexible with the anesthesia plan. Let’s say the resident is suggesting one particular thing and the attending wants to alter it a little bit. I think that’s fine. But if they are too specific about doing it their way, this resident may not be able to experience and see what the plan goes . . . if the other plan is not too much different from the plan that the residents suggest, perhaps the attending can be flexible and let residents do their plan. And, for better or worse, they’ll see the result of their plan and then form their own learning.”
	“The best teachers are actually very open minded. With some attendings, I like to try new things and push the limits. Other attendings are just like they only do one way and they only know how to do it one way and they can't deviate from that. So you have to do it their way and that makes it harder for me to learn. But I want to be able to try new things. And if they're too close minded to new techniques then I don't learn from them very well.”
Practice techniques in non-pressure environment	“Teaching on different ways to approach a difficult airway or using advanced airway devices on patients who may not be difficult electively. I think those are some of the things that we don't really do until we're in an extreme situation. So we hopefully are going to practice in a non-pressured environment.”
Articulation – ask learners to talk through activity, explain their thinking, and or describe their rationale for approach or decision	
Teach back (ask residents to explain what they were taught)	“[An attending] taught me about something and then said, 'OK. Now you teach me what I just taught you.' And I think . . . [I could] retell what I just learned . . . and if you don't do well for some, he’ll teach it again and then he'll ask you to teach it to him again. I think that is a really effective method of learning.”

Verbatim quotes in Table 3 illustrate those codes grouped under social characteristics.

**Table 3:** Social Characteristics of OR Teaching with Verbatim Quotes OR: operating room

Code	Verbatim quote example
Context (teach something relevant to the case)	“The first thing that is very helpful is something contextually relevant . . . [When] something is happening in the OR, or there is some part of the anesthetic technique that we are doing, a medication we are using, or an airway device we are using then the best teachers use that as a springboard to teach something.”
	“Focusing the teaching on obviously what's relevant to the case and what we're doing that day . . . every day we do something different but when it's relevant to the case and relevant to the patient that we're taking care of, then it also helps me as a learner to remember things more and when I think about this issue. I can use this piece of knowledge when I take care of a similar patient with a similar case in the future.”
Commitment (take time to teach, make an effort)	“A really good teacher actually bothers to take the time to teach in the OR at times that are appropriate for patient care.”
	“It's really nice when attendings can take some time during the OR to do some explicit teaching. Have some time with the attendings as opposed to having an attending be there and help us get through the critical moment of the case, but then not be there for the other parts . . . because I think there are some like great teaching opportunities during rest of case.”
Psychological safety (be open and approachable to questions from resident)	“[The best teachers] would be peer available for question. Because sometimes I have questions but if they seem so busy, I will be reluctant to approach them and ask questions.”
	“A good teacher is willing to have that discussion and understand that, not just because questions are being asked it doesn't necessarily mean that I'm questioning their competency. I'm just questioning to better understand the topic.”
	“To have open communication and interaction, is important to be responsive to the teaching, sort of it. If you have kind of wall, it’s a little hard to engage, or to ask question, or set expectations.”
Equanimity (calm and collected)	“The ones that are able to stay calm and collected when things aren't going smoothly or where there is an emergency or something. I've been in a couple rooms where the attendings lost their temper or they've panicked. And it makes a horrendous learning experience.”
	“I find that it's much more effective to be learning while doing when a teacher is calm under pressure. Attendings who are able to stay calm and help us think through the process during induction or emergence, for example, particularly during crises in the OR, they are just better at communication, more effective learning for us than somebody who gets frustrated.”
Proper timing for instruction	“I learn best when I'm not preoccupied by something. So if the patient is unstable or it's the beginning or the end of the case, those are all things that are preoccupying me. So finding a down time in the middle of the case is preferential.”
	“Unfortunately, sometimes the teaching will occur while you're intervening or doing other things. And so, it can be a little distracting and I'm unable to focus entirely on the care of the patient whereas really good teachers are able to come back in after the patient settled and then take a few minutes to teach.”
Belonging (facilitate communication with OR team)	“It’s someone who communicates well . . . it would be with us as residents but also with all the colleagues, the surgeons in the room, and the nursing staff. Because that creates a better and more comfortable environment to work in.”
Confident clinically	“The best ones are clinically confident themselves. Meaning they are comfortable, they don’t freak out over little things and you can just sense they are confident in their ability to adapt to different situations.”
Personable and open	“Just being personable, open and friendly . . . If you feel like you're not really connecting with your attending, it can be an uncomfortable experience. Better when you are working on a team with somebody who supports you and who has your best interests at heart and is going to be there to help when you need them.”
	“I think really good teachers because we're always one on one in the OR usually get to know me personally. So spending just a few minutes to know more about who I am and what my interests are then I feel like I'm more engaged in what they have to offer.”
Ask for feedback for themselves	“The good teachers often seek feedback for improvement, assessing how the day went, what things they can do to improve the experience for the resident, too.”
	“I think when the attendings are open to feedback themselves, too, it makes them more approachable for us as residents and it makes us more open to learning from them. I've had attendings ask me, ‘What can I do better? How do you learn best?’ When they open up that dialogue, I think it makes it more comfortable for the residents.”

One code derived from the resident interviews, focus on a few learning points, was classified into the content theme (Table 4).

**Table 4:** Verbatim Quote Examples of Focus on a Few Learning Points

Code	Verbatim quote examples
Focus on a few learning points	“Having the attending focus on only a couple of things during anesthetic care. If they're continually giving advice on every small aspect of your care, it can be overwhelming. You can actually start to perform under what you'd normally perform as well as get sidetracked off of taking care of the patient's primary. Focus on one or two things per case.”
	“I'd rather focus on two or three learning points for the day and not just overwhelm you with all the nitty-gritty.”

## Discussion

Phenomenology influences research questions by placing emphasis on a phenomenon to be explored, phrased in terms of a single concept or idea [12]. In this study, the phenomenon of interest is OR teaching, as described via interviews with the anesthesia residents. The results of this study affirm the characteristics of good teaching identified as valuable in adult learning theory and that are useful in the OR. The two behaviors most commonly positively recognized by the residents were: 1) autonomy whereby the attending steps back and lets the resident work through patient care in the OR, and 2) reasoning whereby the attending explains the rationale underlying decisions and actions delivered in the care of the anesthetized patient. Social characteristics were also often singled out by residents and related to teaching in the context of the patient at hand and having the attending show effort and commitment to teaching.

We choose to use the cognitive apprenticeship framework because the novice interacting with the expert helps the novice understand the stages needed to become an expert. In contrast to traditional apprenticeship, cognitive apprenticeship emphasizes that learning considerations, not workplace needs, drive the assignment of learner tasks and problems. Teaching emphasizes the application of knowledge. Anesthesia education in the OR lends itself to this framework because of faculty supervision of the resident aimed at the development of knowledge and skills through OR anesthesia practice.

The residents in this study verbalized examples of three of the four cognitive apprenticeship framework themes (teaching strategies, social characteristics, and content) but not sequencing. Sequencing refers to progressively increasing the complexity and diversity of learning activities. An example might be showing proficiency in a low complexity task (intubating a straightforward airway) before moving to higher complexity airway tasks. Sequencing may be less applicable in the OR because of the short duration of the OR day so there may not be enough time for the attending to increase task complexity during the one day. Sequencing also refers to building global conceptual maps before acquiring specific skills. It is also possible that residents didn’t realize or were able to synthesize that some OR teaching involves sequencing.

Residents spoke about several other social characteristics established by successful teachers in the OR. The residents look favorably on attendings who are easily approachable for asking questions (psychological safety) and communicate well with other staff so that the residents can work smoothly and peacefully in the OR (belonging). Residents further asserted that the best attendings stay calm even during difficult cases, were confident in their ability in the OR so could come to the aid of the resident in any situation, and connected well with the resident on a personal level.

A few residents emphasized that attendings should be mindful of proper timing for teaching so that it occurs when the resident is not occupied with the patient and. therefore, can pay attention to what the attending is communicating. This illustrates cognitive load theory, as it is easy to overwhelm the trainee’s limited working memory capacity because of the cognitive processing required to complete a new task or understand a new concept [13]. In other words, if the resident is placing a central line for the first time, not much of what the attending is saying that is not explicitly related to real-time feedback will be remembered by the resident. Limits to the working memory capacity interfere with learning due to different forms of cognitive loads: intrinsic (the level of difficulty of the problem), germane (learning and development of schema), and extraneous (material not relevant to the problem at hand). The attending can try to reduce cognitive overload by providing germane examples, reducing extraneous load, and breaking down complex problems into smaller components.

Finding the proper timing to teach is a unique challenge in the OR. Some days, the attending may, for example, have one room with an all-day case with some invasive lines and a difficult airway. After the case is underway, the attending may have an opportunity to do some explicit teaching during the surgery. In contrast, if the attending is double-covering with several cases in each of the two rooms, and after getting the residents out for breaks, there may be little time left over to do explicit teaching, at least in some of the ways described by some of the residents in this study. During such challenging days, the faculty may find themselves teaching the residents during induction, which may result in cognitive overload because they are trying to concentrate.

Several high-value teaching strategies that were mentioned by the residents related to coaching. Examples included bringing an article relevant to the case to the OR, using a graph or diagram (visualization) to promote a better understanding of complex ideas, discussion as a form of interactive learning, instead of one-way lecture-style learning; and providing immediate feedback while residents are managing patients or right after they finished a procedure (real-time feedback). Online forms can be used to document this type of feedback [14].

High-performing teaching attendings also support and encourage the performance of activities at the resident’s learning edge and with the attending gradually decreasing support over time. These were grouped under scaffolding and were verbalized by the residents in different ways: assess the resident’s specific knowledge level before embarking on a teaching route, challenge residents out of their comfort zone and help them get through that challenge, and ask the resident questions to make the resident think and reflect.

Other desirable techniques identified by the residents were grouped under articulation. Examples are to ask the resident to explain what they just learned so that the attendings can assess understanding and explore whereby the attending is flexible and open to different plans that are possible and which the resident suggests.

The content dimension of the cognitive apprenticeship framework includes factual, conceptual, and procedural knowledge as well as how to use these kinds of knowledge. This expertise includes facts, concepts, procedures, problem-solving, metacognitive, and learning skills. Several residents mentioned the importance of having the attending focus on a few take-home teaching points and not offering so much content as to risk overwhelming the resident.

This study has several limitations. Being a single-institution study, the results may not be generalizable as a different department with different residents may have valued the attributes of good OR anesthesia teaching differently. Other training programs may have a different culture or learning environment within which trainees function [15]. This environment includes the pressures, stresses, rewards, and any other influences perceived by learners [16]. Although the residents interviewed for this study are exposed to more than 150 attendings, the atmosphere, workload, supervision, structure, and organization of other residencies may be sufficiently different so as to make our results less applicable elsewhere [17].

Interviewing more residents may have yielded more data about OR teaching, but after the first 24 residents were interviewed, the additional final residents did not yield any new codes, suggesting that the major views and experiences on the best teaching characteristics were captured.

Also, not enough residents were studied to see if there were real differences in responses, as residents progress through training. Ascertaining any such differences, such as comparing CA3s versus CA1s was not the goal of this study and may deserve attention in future studies. Also, a different group of investigators may have coded the text of the responses differently. For example, we choose to separate “Commitment (take time to teach, make an effort)” from “Literature (bring papers relevant to the case)” even though if an attending brings an article that would be a sign of commitment. Our reasoning was that the two codes were separate specific behaviors that could be explicitly discussed with faculty desiring to improve their OR teaching.

Another limitation is that it is not known whether the practices identified by the residents actually ultimately result in more proficient residents or in better patient outcomes. Learners may not always have insight into what they need to learn and how best to learn it. In fact, though the residents want a related article to the case only on the spot, they did not mention that they want the attendings to discuss it further the next day or later. This may be especially true for less experienced residents, as they may not be able to perceive the value of learning something that experience has taught the faculty is important. An effective teacher may provide context and support for why the teaching point is important, but a resident may discount this. At the K-12 level, studies show that students often fail to identify good teaching techniques such as, for example, evaluating a charismatic teacher who lectures didactically better than a more subdued teacher that implements active learning principles. In contrast, residents may be more mature and understand what teaching benefits their learning.

In post-graduate medical education, learning occurs through the accumulation and processing of clinical experiences. As residents participate in clinical work, learners have a variety of sources of information, or ‘learning cues,’ that facilitate the interpretation of the experience and the construction of expertise. In this setting, teaching is a multifaceted undertaking [18]. Even though 27 different behaviors and characteristics were identified, none of the 27 was mentioned by more than 13 of the 30 residents interviewed. This suggests that there exists a wide range of activities that go into optimal OR teaching. Leveraging the knowledge of medicine and patients with knowledge of context and pedagogy and learners into teaching scripts can guide the faculty to choose optimal content and teaching interactions [19].

## Conclusions

Best practice for OR teaching, as perceived by anesthesia residents, includes social characteristics, such as context, commitment, psychological safety, equanimity, and proper timing, as well as teaching methods, such as autonomy, reasoning, literature, prior knowledge, flexibility, reflection, real-time feedback, and teach back. Further studies can determine if training the anesthesiology faculty to incorporate these elements increases the caliber of daily teaching in the OR.
